# Optofluidic laser speckle image decorrelation analysis for the assessment of red blood cell storage

**DOI:** 10.1371/journal.pone.0224036

**Published:** 2019-10-22

**Authors:** Hee-Jae Jeon, Muhammad Mohsin Qureshi, Seung Yeob Lee, Euiheon Chung

**Affiliations:** 1 Department of Biomedical Science and Engineering, Gwangju Institute of Science and Technology (GIST), Gwangju, Korea; 2 Department of Laboratory Medicine, Chonnam National University Hospital, Gwangju, Korea; Universitat Zurich, SWITZERLAND

## Abstract

Red blood cells (RBCs) undergo irreversible biochemical and morphological changes during storage, contributing to the hemorheological changes of stored RBCs, which causes deterioration of microvascular perfusion in vivo. In this study, a home-built optofluidic system for laser speckle imaging of flowing stored RBCs through a transparent microfluidic channel was employed. The speckle decorrelation time (SDT) provides a quantitative measure of RBC changes, including aggregation in the microchannel. The SDT and relative light transmission intensity of the stored RBCs were monitored for 42 days. In addition, correlations between the decorrelation time, RBC flow speed through the channel, and relative light transmission intensity were obtained. The SDT of stored RBCs increased as the storage duration increased. The SDTs of the RBCs stored for 21 days did not significantly change. However, for the RBCs stored for over 35 days, the SDT increased significantly from 1.26 ± 0.27 ms to 6.12 ± 1.98 ms. In addition, we measured the relative light transmission intensity and RBC flow speed. As the RBC storage time increased, the relative light transmission intensity increased, whereas the RBC flow speed decreased in the microchannel. The optofluidic laser speckle image decorrelation time provides a quantitative measure of assessing the RBC condition during storage. Laser speckle image decorrelation analysis may serve as a convenient assay to monitor the property changes of stored RBCs.

## Introduction

Red blood cells (RBCs) are the most widely transfused blood component, frequently required for patients with anemia or significant blood loss [1, 2]. RBCs can be stored for up to 35 days within an anticoagulant solution (typically CPDA-1; citrate-phosphate-dextrose-adenine-one) at 4 ± 2°C depending on the condition of preservation [3, 4]. During storage, RBCs undergo a series of biochemical and mechanical/morphological changes, including RBC deformability, hemolysis, and aggregability. These changes may cause negative effects known as ‘storage lesions’ on RBC transfusion [5–8]. In particular, RBC aggregability may contribute to microcirculatory impairment, causing risk for the recipients [9]. Blood storage can alter the hemorheological properties due to the changes of RBC deformability and aggregability [9–11]. Thus, further researches are necessary to understand the rheological changes in stored blood for clinical application [11, 12]. Firstly, micropipette aspiration was used to investigate the stored RBC conditions, using negative pressure to aspirate the RBCs into the micropipette. Recently, stored RBC conditions have been characterized by stretching RBCs using a rotating plate under certain shear stress [13]. Not only has the optical trapping force been adopted to check the stored RBC condition by stretching the RBC, but also a microfluidics measurement using dielectrophoresis force has been suggested to investigate the stored RBC condition [14]. However, these techniques may not be capable of checking large volumes of RBCs continuously, and they are time-consuming and expensive.

In this study, we report a novel approach to monitor the properties of stored RBCs with a measurement of the speckle decorrelation time (STD) of the RBCs flowing through a microfluidic channel. We developed a highly sensitive speckle intensity fluctuation analysis in an optofluidic system and investigated whether the speckle intensity fluctuation in stored RBCs could be used to evaluate the storage-dependent RBC property change under cold storage conditions.

## Materials and methods

### Theory

The laser speckle patterns of the interference of light after multiple scattering through a medium containing dynamic scatters can fluctuate over time. The effect of RBC property changes including aggregation can be probed by monitoring the fluctuation of the speckle pattern from an optically transparent microfluidic channel over time. There are two standard approaches to calculate the autocorrelation function. The first approach includes an analysis of the power spectrum of the detected signal:

$$S(\omega )=\int I^{2}(t)\cos\left(\omega t\right)\;dt$$ (1)

For the second, the temporal autocorrelation of the intensity can be written as

$$g_{2}(\tau )=\frac{\sum_{m}I_{m}(t_{0})I_{m}(t_{0}+\tau )}{M}\frac{\sum_{m}I_{m}(t_{0})}{\sum_{m}I_{m}(t_{0}+\tau )}-1,$$ (2)

where *I*_*m*_(*t*) is the transmitted scattered light intensity captured by the detector at time *t*_*o*_ and *t*_*o*_+*τ*, while *M* is the total number of pixels on the sensor. The intensity and electrical autocorrelation function can be converted into the field autocorrelation function by the Siegert relationship [15].

$$g_{2}(\tau )=1+{\beta |g_{1}(\tau )|}^{2}$$ (3)

Here, *β* depends on the laser coherence length, laser stability, and the number of the speckles detected. In our experiment, *β* was determined to be 0.6 ~ 0.7 by calculating *β* = *g*_2_(0)−1. *g*_1_(*τ*) is the electrical field autocorrelation function that can be written as [15, 16]:

$$g_{1}(\tau )=\int_{0}^{\infty }P(s)exp\;[(-\frac{2\tau }{\tau_{o}})\frac{s}{l^{*}}]\;ds$$ (4)

However, by examining different shear flow depending on an intensity-based autocorrelation function, with reference to Wu et al., Eq (4) becomes [17]:

$$g_{1}(\tau )=\int_{0}^{\infty }P(s)exp\;[-2[\tau /\tau_{B}+{\left(\tau /\tau_{s}\right)}^{2}]\frac{s}{l^{*}}]\;ds$$ (5)

where ${\tau_{s}}^{-1}=\Gamma k_{0}l^{*}/\sqrt{30}$ and *τ*_*B*_ = *D k*_0_^2^ are the relaxation time from laminar shear flow and Brownian motion, respectively, Γ is the shear flow, *P*(*s*) is the particle distribution of path lengths in the medium, *τ*_*o*_ = 1/(D*k*_0_^2^) is the mean-square displacement of the scattering particle characterized decay time, *k*_0_ is the wavenumber of the light in the medium, *D* is the diffusion coefficient, l* is the transport mean-free path, and *s* is the path length [16, 17]. From this equation *P*(*s*),*k*_0_, and l* are essentially fixed in the same experimental configuration. Additionally, from this equation, it is expected that *g*_1_(*τ*) will decay faster by increasing the shear flow (*Γ*). The diffusion coefficient can be described by the Einstein-Stoke equation:

$$D=\frac{K_{B}T}{6\pi \eta r}$$ (6)

where *K*_*B*_ is Boltzmann’s constant, *T* is the absolute temperature, *η* is the viscosity of the medium, and *r* is the particle radius. *g*_1_(*τ*) also decays more rapidly for smaller particles and lower viscosity. According to the theory, the SDT *g*_1_(*τ*)) will vary depending on medium viscosity (*η*) and particle size (*r*). Furthermore, plasma viscosity, RBC deformability, and aggregation were well known to increase with increasing RBC storage period which shows and explain the change in viscosity of stored blood with the storage time [18, 19]. Because the effective particle size may be bigger with stored RBC aggregation, we measured the SDT of stored RBCs based on the autocorrelation function in a microfluidics chip with a square shape.

### Speckle fluctuation measurement system

**Fig 1:**
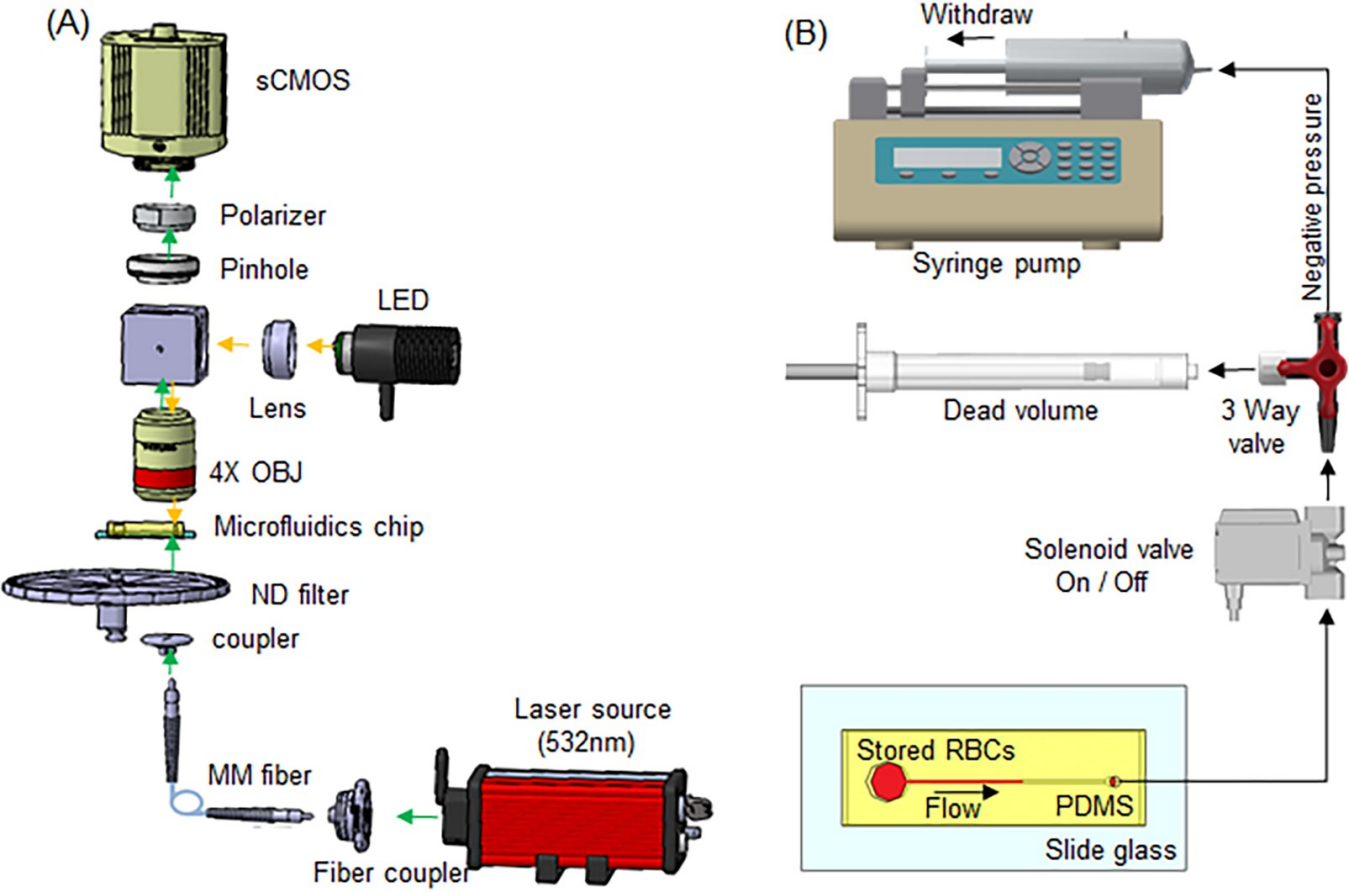
Setup for measurement of RBC decorrelation time with a microfluidic channel system for stored RBCs. (A) Optical experimental setup used to capture speckle intensity fluctuation; (B) Microfluidic system for flowing RBCs through a transparent microchannel comprising a microfluidics chip and vacuum flow generation. (LED: Light emitting diode, MM fiber: multimode fiber, ND filter: Neutral density filter, OBJ: Objective lens, PDMS: Polydimethylsiloxane).

We captured the RBC speckle intensity fluctuation with respect to RBC storage duration and measured the SDT using a home-built laser speckle imaging system with a microfluidic channel (Fig 1). An optical table with vibration isolation was used to ensure the stability of laser speckles with minimum noise. A diode-pumped solid-state (DPSS) laser (𝜆 = 532 nm) with an output of 50 mW (LSR-0532, Changchun New Industries Optoelectronics Technology Co., Ltd., China) was used to transilluminate the microfluidic channel from a multimode fiber (M72L02, Thorlabs, New Jersey, USA) connected to a fiber coupler (Thorlabs PFA-X-2-B). The microfluidic chip was gently fixed with a slice anchor (Warner Instruments, SHD-22L/15) at room temperature (20°C ~ 22°C). The laser was turned on for 5 min to stabilize the light source before conducting experiments (S3 Fig). The light speckle intensity fluctuation was captured using a microscope objective lens (Plan N 4C, NA 0.1, Olympus) focused on the top surface of blood flow in the microchannel, together with a linear polarizer and an aperture (diameter: 8 mm) to match the size of a single speckle to approximately 3×3 pixels. We used a scientific CMOS camera (Neo 5.5 sCMOS, Andor Technology Ltd. Belfast, UK) at a frame rate of 1,250 frames per second with an exposure time of 0.8 ms.

### Blood collection from rat tail

The experiment was carried out using male Sprague Dawley rats (*n* = 13, 14~16 weeks old with body weights between 280 and 300 g). This animal handling was carried out in strict accordance with the recommendations in the guidelines of the Institute Animal Care and Use Committee of the Gwangju Institute of Science and Technology, South Korea. The protocol was approved by the Laboratory Animal Resource Center, Gwanju Institute of Science and Technology, South Korea (Protocol Number: GIST-2019-015). All procedure was performed under gas anesthesia, and all efforts were made to minimize suffering. The tail vein was used to draw 1.5 ~ 2 ml of whole blood using a 26 G needle from animals. The blood was stored in a sterile blood collection tube with citrate-phosphate-dextrose-adenine (CPDA-1) [20]. Platelet-free plasma was separated by centrifugation at 3000 G for 20 min followed by removal of the platelet-rich plasma region. The RBCs were washed three times with isotonic phosphate buffered saline (PBS, pH +7.4, 290 mOsmol/kg). The RBCs were then resuspended in the platelet-free plasma. The RBC samples acquired from each animal (n = 13) were aliquoted and kept in the refrigerator until experiments. For all our experiments, hematocrit was fixed at approximately 45%. Each measurement was performed with different aliquot with a volume of 100 μl which was taken out of the refrigerator and placed in the inlet chamber. The treated RBCs was exposed to the room temperature for five minutes prior to the experiment to reach thermal equilibrium with room condition.

### Fabrication of microfluidic devices

The microfluidic devices were fabricated with standard soft photolithographic techniques. Master mold fabrication includes a spin coating of a photoresist film, and exposure mask to UV light, and developing a pattern on a silicon substrate. We attached the completed master as a mold on a petri dish and poured the polydimethylsiloxane (PDMS; Sylgard 184 A/B, Dow Corning, South Korea) mixed with a based and curing agent. Petri dish with PDMS inserted on a vacuum chamber for 30 minutes to remove air and cured in an oven (80°C for 1 hour). After punching holes (inlet 5 mm, outlet 1 mm diameter) with biopsy punches, we treated the PDMS slab with plasma at 250 W and 80 m Torr (CUTE, Femto Science Co., Korea) for 50 seconds. After oxygen plasma treatment, the PDMS slab was bonded to the slide glass [17, 21].

### Microfluidics system operation

The microfluidics system was made up of a vacuum generator consisting of a syringe pump in withdrawal mode, a solenoid valve, a 3-way valve, and a 50 ml syringe for dead volume. The microfluidic chip contained a channel of 45 mm in length, 1 mm in width, and 45 μm in height. The region of interest (ROI) for the speckle imaging (128 × 512 pixels) was chosen near the outlet reservoir with a 1 mm diameter at the end of the channel sides, as shown in S4 Fig. The treated-blood sample was introduced to the inlet of the microfluidics chip. We used 1 mL syringe racked in the syringe pump (Pump 11 Elite Programmable Syringe Pump, Harvard Apparatus, US), the pulling volume was fixed at 200 μL using a withrawal flow rate of 5 mL/min. When the solenoid valve was opened, the treated-blood samples were driven by a negative pressure gradient inside the microchamber. We applied a relatively large dead volume chamber, which was connected to a syringe pump to relieve pressure fluctuation during the experiment. The treated blood began to flow into the microfluidics channel under a constant pressure gradient.

### Speckle decorrelation time (SDT) analysis

The 1,250 images—more than enough to obtain a complete SDT obtained with the Andor camera—were stacked and stored on a .tif file format using the Andor software, Solaris S. The images were analyzed with a custom-written Matlab program to compute the decorrelation time which took a few seconds. (Intel single core i7-6700). The autocorrelation function, known as the serial correlation per image, of the intensity distribution of pixels with time was calculated. The space-time correlation is described by Eq 2. The decorrelation time was calculated as the time for a correlation between the initial image and the subsequently captured images of the speckle time series. We took the half value of the autocorrelation curve to calculate the decorrelation time $\left(i.e.,\frac{Initial\;value\;g_{1}(\tau )+Last\;value\;g_{1}(\tau )}{2}\right)$ [22]

### Relative light transmission intensity and RBC flow speed analysis

The relative light transmission intensity and RBC flow speed were recorded simultaneously. The RBC flow within the ROI was calculated as the transit time of the blood from the start to the end of the ROI of 0.8 mm. Simultaneously, the light transmission ratio was measured as the treated RBCs reached the end of the ROI region (S1 and S2 Figs). As the blood reached the end of the ROI region, the light speckle pattern was recorded for 2 s using the sCMOS camera. The resultant RBC flow speed and light transmission versus time profiles were analyzed to determine the magnitude and time course of aggregation [23–25].

## Results

### SDT varies with RBC storage duration

**Fig 2:**
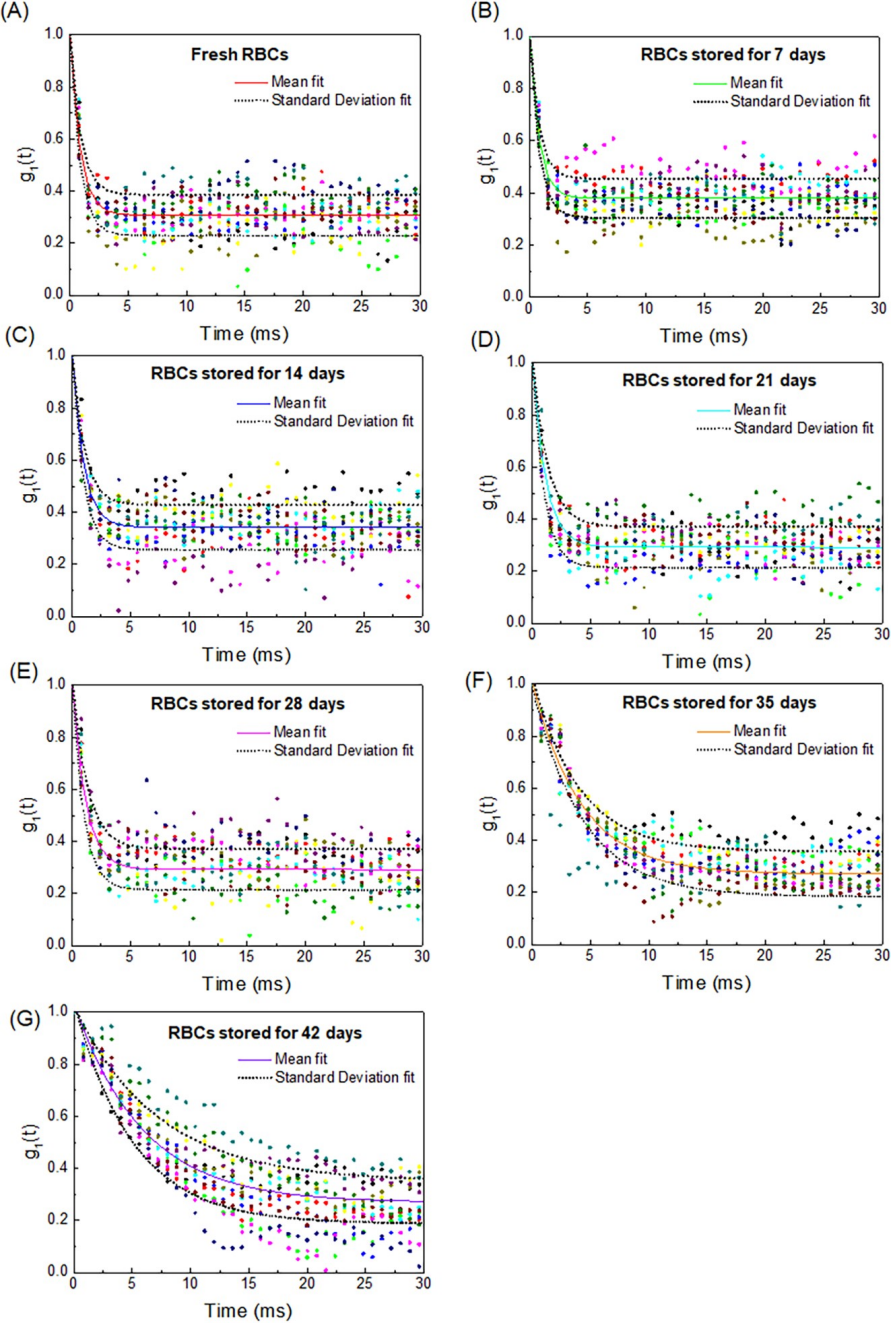
Speckle correlation curves for fresh RBCs and stored RBCs on days 7, 14, 21, 28, 35, and 42 of storage. The center of the bold line with different colors shows the double exponential curve fitting from the sample mean value while the two outer dotted lines indicate the curve fitting from the sample standard deviation. Data points are shown for 13 datasets of fresh RBCs and stored RBCs for 7, 14, 21, 28, 35, and 42 days (A ~ G). The mean decorrelation curve is overlaid as a continuous line (*n* = 13).

**Fig 3:**
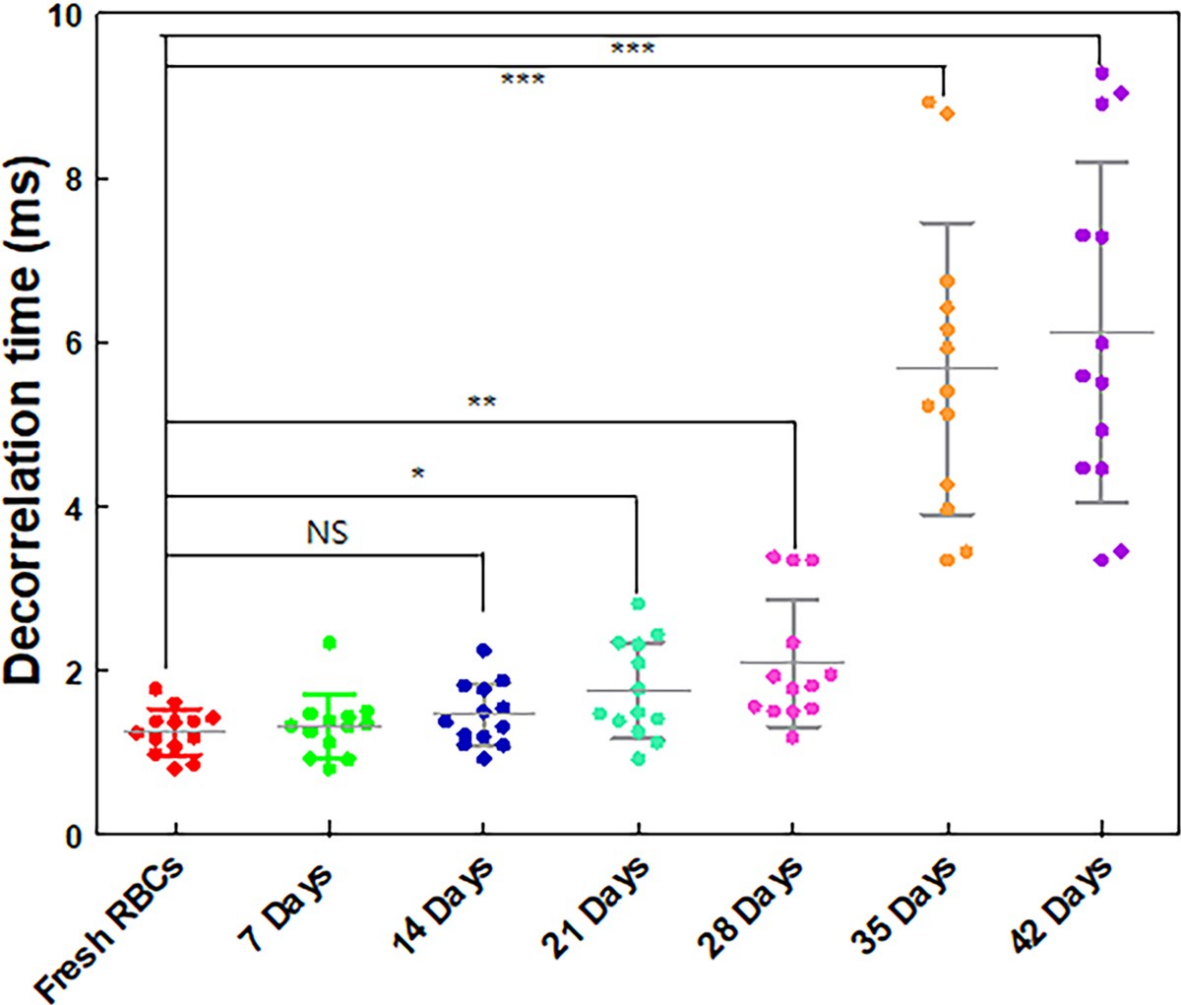
Speckle decorrelation times (SDTs) of stored RBCs with respect to storage duration. The decorrelation times of each group were 1.26 ± 0.27 ms, 1.32 ± 0.36 ms, 1.47 ± 0.37 ms, 1.76 ± 0.56 ms, 2.10 ± 0.74 ms, 5.68 ± 1.71 ms, and 6.12 ± 1.99 ms for fresh RBCs, stored for 7 days, 14 days, 21days, 28 days, 35 days, and 42 days duration repectively. (n = 13), NS: no significant difference, *: p < 0.05 and ** 0.05< p < 0.001, ***: p < 0.001.

**Fig 4:**
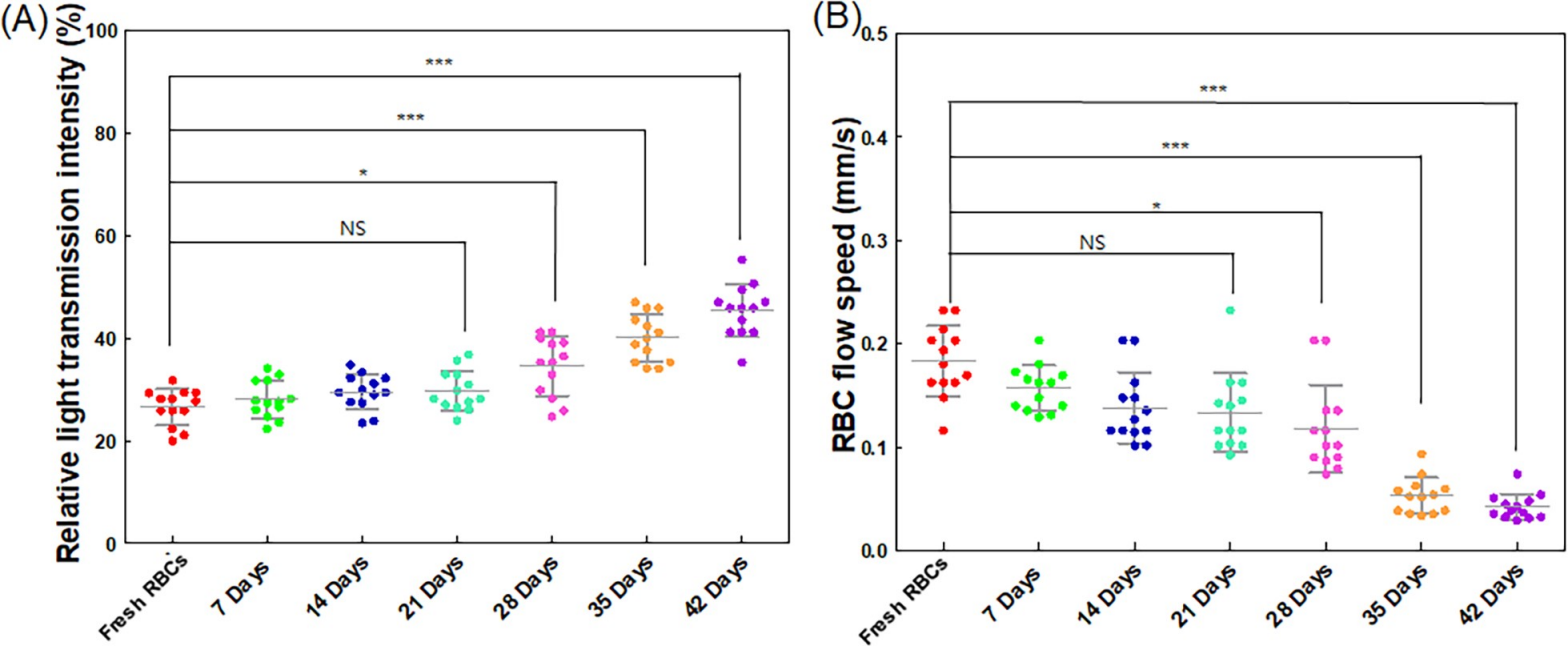
Relative light transmission intensity and RBC flow speed analysis with respect to storage duration. (A) Relative light transmission intensities of stored RBCs with respect to storage duration. The relative light transmission intensity was calculated from $\left(\frac{l_{t}-l_{b}}{l_{b}}\right)\times 100$ after acquired a series of speckle image. (*l*_*t*_: transmitted light intensity, *l*_*b*_: background light intensity); (B) RBC flow speeds of stored RBCs in the region of interest (ROI). The speeds were measured from the ROI starting position to the end of the channel in the ROI for stored RBCs (*n* = 13). NS: no significant difference, *: *p* < 0.05 and ** *0*.*05< p* < 0.001, ***: *p* < 0.001, *l*_*t*_ is the light transmission intensity, and *l*_*b*_ is the background intensity.

We measured the SDT for stored RBCs for 6 weeks. RBCs were stored at 4°C in a refrigerator. The RBC decorrelation time was evaluated on the first day of blood collection and was repeated on days 7, 14, 21, 28, 35, and 42 of storage (*n* = 13 animals). The speckle correlation curves were analyzed with double exponentials depending on the RBC storage duration (Fig 2). Each SDT was measured at half of the initial and the last values [22, 26]. The SDT increase was more prominent after 28 days of RBC storage (Figs 3 and 4).

We compared the SDTs with respect to the duration of RBC storage (Fig 3 and S1 Movie). While the SDT of the RBCs stored for up to 14 days was not significantly different from that of the fresh RBCs, the SDT increased after 21 days. Notably, the SDTs significantly increased after 28 days. We noted differences in the decorrelation times of RBCs for different RBC storage durations (Fig 3).

We also measured the transmission light through the channel and analyzed the relative light transmission intensity change depending on RBC storage duration. The trend was similar; the light transmission was increased over the RBC storage duration (Fig 4A) which is mainly associated with RBC aggregation [27–29]. The transmission light intensity was background subtracted and the differences were normalized with the background intensity. To measure the background intensity, the channel was kept empty, and speckle images were captured through transmission light intensity passing through the channel. The relative light transmission intensity of the stored RBCs were 26.57 ± 3.39 (%), 28.08 ± 3.49 (%), 29.54 ± 3.25 (%), 29.76 ± 3.72 (%), 34.54 ± 5.54 (%), 40.09 ± 4.46 (%), and 45.34 ± 4.85 (%) for the corresponding durations of 0 (fresh RBCs), 7, 14, 21, 28, 35, and 42 days, respectively. The low relative light transmission intensity indicates less RBC aggregation while the higher relative light transmission intensity shows more RBC aggregation. In addition, we measured the RBC flow speed through a defined ROI near the end of the microfluidics channel (The center of rectangular ROI (0.8 mm × 2 mm) was located at a distance of 42 mm from the inlet and 0.5 mm from the sidewall (shown in S4 Fig)). As the RBC storage duration increased, the migration speed of the RBC sample slowed (S2 Movie). Fig 4B shows the RBC flow speed depending on the storage day. The RBC flow speeds were 0.18 ± 0.03 mm/s, 0.16 ± 0.02 mm/s, 0.14 ± 0.04 mm/s, 0.13 ± 0.04 mm/s, 0.11 ± 0.04 mm/s, 0.05 ± 0.016 mm/s, and 0.042 ± 0.011 mm/s for the corresponding durations of 0 (fresh RBCs), 7, 14, 21, 28, 35, and 42 days, respectively.

**Fig 5:**
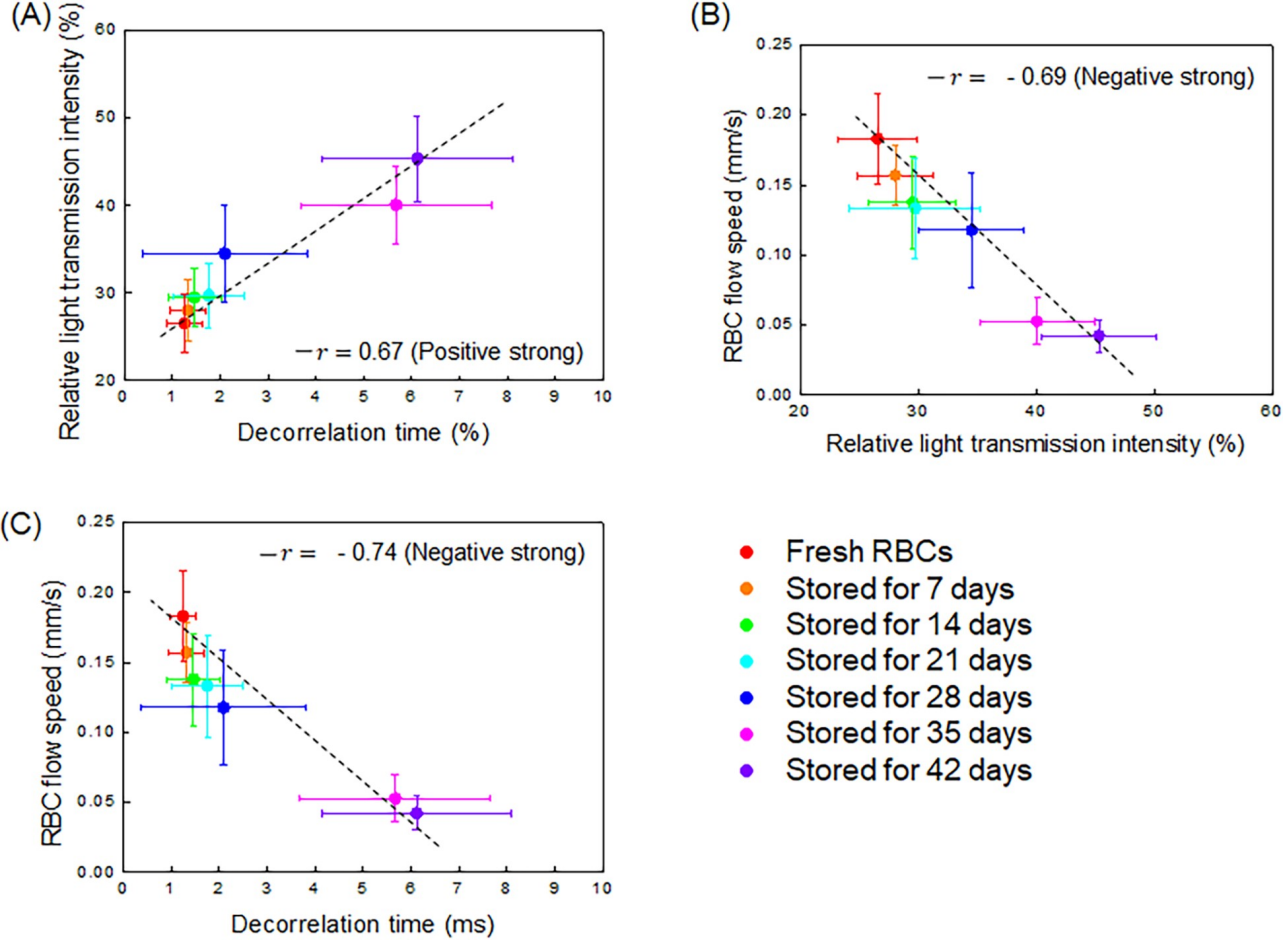
Averaged correlation plots and map among relative light transmission intensity, RBC flow speed, and decorrelation time over the RBC storage duration (n = 13). The relationships between (A) relative light transmission intensity and decorrelation time, (B) RBC flow speed and relative light transmission intensity, (C) RBC flow speed and decorrelation time. The solid line indicates the linear correlation coefficient of the data points. The closer r is to 1 or –1, the more linear the scatterplot of each group’s variables (-1≤r≤1).

To investigate the correlations between the speckle decorrelation time, RBC flow speed and relative light transmission intensity, we plotted three correlations and computed the corresponding linear regression lines based on the equation y = *a*_0_+*b*_0_*x* (linear nonhomogeneous function), as shown in Fig 5. The RBC flow speed has moderately negative correlations with both the relative light transmission intensity and SDT (*r* = –0.69, and *r* = –0.74, respectively, Fig 5B and 5C. The relative light transmission intensity and SDT have a positive linear correlation with *r* = 0.67).

## Discussion

It has been observed that stored RBCs undergo irreversible changes in their biomechanical and biochemical properties, and many researchers suggest these changes occur during RBC storage [30–32]. The routine temperature for cold storage is usually 2–5°C; because of this, RBC aggregation increases as a result of oxidative injury [30]. The aggregation level increase in stored RBCs is a major determinant of blood viscosity as it reduces the flow speed gradually due to the hydrodynamic resistance during the blood migration [18, 33]. According to Wu et al., the SDT increases as the particle size (*r*) in the fluid increases, and we consider a similar relationship between the SDT and aggregated RBCs in the stored sample [17]. The SDT also depends on the viscosity (*η*); the more viscous the fluid, the longer is the SDT we obtain. However, according to Eq (5), the speckle decorrelates faster, i.e., SDT reduces, as the shear flow increases [17, 34]. Therefore, we can conclude that larger particle size (*r*) and high viscosity (*η*) are the reasons for a slower SDT, while increasing the shear flow results in a faster SDT of stored RBCs.

In this study, we determined the SDT of fresh RBCs and stored RBCs for a duration of up to 42 days, and we observed that SDT increases as the stored RBC duration increases. The SDT of stored RBCs for longer periods (28 ~ 42 days) increased significantly compared with that of the RBCs stored for 21 days and shorter, as shown in Fig 3. The SDT of stored RBCs, fresh ~ 21 days, appeared to slightly increase without statistical significance. It was reported that the significant depletion of ATP degradation and oxidative injury increased more after 28 days of storage, therefore, the aggregation level of the sample increased [35]. Furthermore, it was observed that RBCs became stiffer after 28 days of storage [36]. These results agree with our findings that the SDT increased after 28 days of storage.

The aggregation of RBCs results in a decreased number of particles in suspension, therefore, the increased light transmission and decreased backscattering is an important factor of in vivo blood flow dynamic [37, 38]. Our data also shows that the relative light transmission intensity increased after 28 days of RBC storage, as shown in Fig 4A. The RBC flow speed dramatically decreased after 28 days of RBC storage, demonstrating an inverse relationship with the SDT. Other studies of relative light transmission intensity also show a similar tendency [10, 11, 27, 35, 38, 39]. However, the level of increase in the relative light transmission intensity and decrease in the RBC flow speed during storage vary among different studies, potentially due to differences in RBC storage methods and different measurement techniques to determine the level of transmission light intensity and RBC flow speed.

In this study, we investigated the relationship of RBC storage duration with SDT, relative light transmission intensity, and RBC flow speed. As shown in Fig 5A, the relative light transmission intensity has a positive correlation with the SDT, which reflects that the RBCs aggregate over a long storage duration resulting in a higher decorrelation time, hence, the relative light transmission intensity increases for these samples [38, 40]. Therefore, the correlation between the relative light transmission intensity and the SDT accounts for the enhanced RBC aggregation in the ROI of the channel. Thus, the increase of sample aggregation not only increases the light transmission intensity, but also the SDT, as mentioned in Eq (5) [17, 40]. We have demonstrated these relationships through the experiments. In contrast, the correlation between the RBC flow speed with relative light transmission and decorrelation time is strongly negative, as shown in Fig 5B and 5C. The reduction in the RBC flow speed with storage duration could be explained by the increased viscosity of the RBC sample, because of the well-established relationship between the viscosity of the RBC sample, RBC aggreagation, deformability, and RBC storage duration [18, 19, 41]. However, the relation between the scale factor and SDT may not be thoroughly explained by RBC aggregation and viscosity only. We found SDT increases with particle sizes and decreases with flow rate using microsphere particles to demonstrate the sensitivity of our approach (S6 Fig).

Our work, for the first time, measured RBC SDT changes during storage by the aggregation of RBCs. We suggest that measurement of the decorrelation time can be exploited to determine the expected duration of RBC storage. Traditional techniques to measure the characteristics of stored RBCs have been investigated, including several techniques for the quantification of RBC aggregation processes, such as erythrocyte sedimentation rate (ESR), image analysis method, ultrasound back-scattering techniques, photometric method, LORCA (mechatronics, Netherlands), and light-transmission slit rheometer (LTSR) [42, 43]. Although these methods have their advantages, they are systematically complicated, e.g., the analysis methods and measurement procedures require large sample volumes. The proposed SDT method involves a simplified system of easy operation with only a low blood volume (10 uL). In addition, our system can offer multiple parameters simultaneously through a single microfluidic device: SDT, relative light transmission, and RBC flow speed, which can provide more precise indicators before the transfusion of blood for patients. The results of our study are in accordance with previous studies investigating RBC aggregation during storage [31, 36, 44].

The current study does have limitations. While the experiments were performed at room temperature (20°C ~ 22°C), a more accurate temperature-controlled environment could have improved the repeatability of our measurements. We used a tube with additional CPDA-1 instead of a clinical RBC storage bag to store the RBCs due to the small volume of rodent blood. Therefore, the quantitative result may not be the same as that of a clinical sample in a blood collection bag. In addition, macromolecules of RBC membrane surface related to blood coagulation and aggregation were affected by cold storage [43, 45, 46]. Thus, the relationship between the macromolecular changes on the RBC membrane surface and SDT is still unknown and worth further study using conventional membrane staining techniques and flow cytometry. Yet, the relationship between SDT and rheological properties of RBC (i.e. RBC deformability, aggregability,) with respect to RBC storage duration is unknown and worth additional investigation (See S5 Fig). Further mechanistic investigation between the SDT and indication of the RBC aging process, including mechanical or biochemical alterations, would provide more insight into the phenomenon. As this proof-of-concept study was conducted using rodent blood, SDT measurement of human blood samples with further characterization would prove the applicability of the SDT in clinical settings.

## Conclusion

In this paper, we demonstrated the first optofluidic laser speckle image decorrelation time analysis to assess stored RBCs with simultaneous measurements of the SDT, transmission light intensity, and blood flow speed in the microfluidic channel. As the stored RBC ages, the decorrelation time was found to increase significantly along with the other measured parameters, indicating that the SDT might be a useful parameter to assess the quality of stored RBC in transfusion medicine. This SDT measurement requires only ten microliters of blood sample and allows a rapid assessment of stored RBC quality in a quantitative manner. This new approach could shed light on the assessment of stored RBCs and help develop better strategies to improve the currently limited RBC storage in transfusion medicine.

## Supporting information

S1 Movie Real-time display of speckle fluctuations of fresh RBCs and RBCs stored for 35 days. (MP4)

S2 Movie Real-time display for RBC flow measurement of fresh RBCs and RBCs stored for 35 days. (MP4)

S1 Fig Detailed flow chart of the procedure used to measure the speckle decorrelation time. The sequence of speckle images over time was used for calculating the field autocorrelation function *(g*_1_(*τ*)*)*. Decorrelation time is defined as the time for a correlation between the initial image and the subsequently captured image to drop by 50%.
(TIF)

S2 Fig Schematic design to measure the relative light transmission intensity and RBC flow speed. (A) shows the schematic for measuring relative light transmission intensity (l_i_ is initial light intensity, l_t_ shows the transmitted light intensity passing through the channel filed with blood sample, and l_b_ is the background light intensity passing through the channel unfilled with samples. (B) RBC flow speed was calculated using average speed v = S_n_/T_n_.
(TIF)

S3 Fig The stability of diode-pumped solid-state laser power output. The schematic of setup to measure laser power fluctuations over time (A) and the acquired data (B).
(TIF)

S4 Fig Schematic of the microfluidic channel, physical dimensions of simple microfluidic chip design, and region of interest (ROI) used to capture a series of speckle images. (TIF)

S5 FigDeformability measurement of stored RBCs.(A) Schematic diagram of the microfluidic device to measure RBC deformation index (DI). Individual RBCs are flowing through the channel undergoing shape changes. (B) RBC deformation indices over storage duration. For measurement of the stored RBC deformability, microfluidic devices was prepared through a typical photolithographic process. Treated blood sample of RBC suspension with 1% hematocrit, in PBS-albumin buffer, was inserted into the flow chamber. Image sequences of RBCs in the channel were obtained by a high-speed camera (Neo 5.5 sCMOS, Andor Technology Ltd. Belfast, UK). The deformation index (DI = $\frac{L-D}{L+D}$) was calculated from the image sequences (L = axial RBC length, D = lateral RBC lengh). DI distribution decreased over the duration of RBC cold storage. High DI shows the highly deformable cell as close to 1, and low DI shows the low deformability. *: p < 0.05 and ** p < 0.01, ***: p < 0.001.(TIF)

S6 Fig Experimental results of decorrelation time using field autocorrelation function measured the variation of microsphere particle (r = particle size) and flow rate (Q) (n = 5). In Figure (A) and (C,) field autocorrelation function curves were calculated by capturing each speckle sequence by comparing the original reference frame. (B) shows decorrelation time was changing from different particle size. (C) show decorrelation time changing from the different flow rate. Particles concentration was prepared from diluted deionized water 3:10 (30% of particle with 1 μm = 12.1 × 109 particles/mL. Each groups decorrelation time were 1.10 ± 0.18 ms, 1.32 ± 0.14 ms and 1.41 ± 0.19 ms respectively in figure(B) and 1.61 ± 0.30 ms, 1.28 ± 0.13 ms, and 1.04 ± 0.21 ms in figure (D).
(TIF)

## References

[1] Vincent JL, Baron J-F, Reinhart K, Gattinoni L, Thijs L, Webb A, et al Anemia and blood transfusion in critically ill patients. Jama. 2002;288(12):1499–507. 10.1001/jama.288.12.1499 12243637

[2] Schmied H, Reiter A, Kurz A, Sessler D, Kozek S. Mild hypothermia increases blood loss and transfusion requirements during total hip arthroplasty. The Lancet. 1996;347(8997):289–92. 10.1016/s0140-6736(96)90466-3 8569362

[3] Moore G, Peck C, Sohmer P, Zuck T. Some properties of blood stored in anticoagulant CPDA‐1 solution. A brief summary. Transfusion. 1981;21(2):135–7. 10.1046/j.1537-2995.1981.21281178147.x 7222197

[4] Gilson CR, Kraus TS, Hod EA, Hendrickson JE, Spitalnik SL, Hillyer CD, et al A novel mouse model of red blood cell storage and posttransfusion in vivo survival. Transfusion. 2009;49(8):1546–53. 10.1111/j.1537-2995.2009.02173.x 19573176 PMC2888981

[5] Blasi B, D’alessandro A, Ramundo N, Zolla L. Red blood cell storage and cell morphology. Transfusion medicine. 2012;22(2):90–6. 10.1111/j.1365-3148.2012.01139.x 22394111

[6] Kim‐Shapiro DB, Lee J, Gladwin MT. Storage lesion: role of red blood cell breakdown. Transfusion. 2011;51(4):844–51. 10.1111/j.1537-2995.2011.03100.x 21496045 PMC3080238

[7] Bennett-Guerrero E, Veldman TH, Doctor A, Telen MJ, Ortel TL, Reid TS, et al Evolution of adverse changes in stored RBCs. Proceedings of the National Academy of Sciences. 2007;104(43):17063–8. 10.1073/pnas.0708160104 PMC2040393 17940021

[8] Kor DJ, Van Buskirk CM, Gajic O. Red blood cell storage lesion. Bosnian journal of basic medical sciences. 2009;9(Suppl 1):S21. 10.17305/bjbms.2009.2750 PMC5655167 19912115

[9] Barshtein G, Manny N, Yedgar S. Circulatory risk in the transfusion of red blood cells with impaired flow properties induced by storage. Transfusion medicine reviews. 2011;25(1):24–35. 10.1016/j.tmrv.2010.08.004 21134624

[10] Nagaprasad V, Singh M. Sequential analysis of the influence of blood storage on aggregation, deformability and shape parameters of erythrocytes. Clinical hemorheology and microcirculation. 1998;18(4):273–84. 9741668

[11] Relevy H, Koshkaryev A, Manny N, Yedgar S, Barshtein G. Blood banking–induced alteration of red blood cell flow properties. Transfusion. 2008;48(1):136–46. 10.1111/j.1537-2995.2007.01491.x 17900281

[12] Uyuklu M, Cengiz M, Ulker P, Hever T, Tripette J, Connes P, et al Effects of storage duration and temperature of human blood on red cell deformability and aggregation. Clinical hemorheology and microcirculation. 2009;41(4):269–78. 10.3233/CH-2009-1178 19318720

[13] Kim G, Lee M, Youn S, Lee E, Kwon D, Shin J, et al Measurements of three-dimensional refractive index tomography and membrane deformability of live erythrocytes from Pelophylax nigromaculatus. Scientific reports. 2018;8(1):9192 10.1038/s41598-018-25886-8 29907826 PMC6003953

[14] Jeon H-J, Lee H, Yoon DS, Kim B-M. Dielectrophoretic force measurement of red blood cells exposed to oxidative stress using optical tweezers and a microfluidic chip. Biomedical engineering letters. 2017;7(4):317–23. 10.1007/s13534-017-0041-4 30603182 PMC6208507

[15] Jang M, Ruan H, Vellekoop IM, Judkewitz B, Chung E, Yang C. Relation between speckle decorrelation and optical phase conjugation (OPC)-based turbidity suppression through dynamic scattering media: a study on in vivo mouse skin. Biomedical optics express. 2015;6(1):72–85. 10.1364/BOE.6.000072 25657876 PMC4317115

[16] Qureshi MM, Brake J, Jeon H-J, Ruan H, Liu Y, Safi AM, et al In vivo study of optical speckle decorrelation time across depths in the mouse brain. Biomedical optics express. 2017;8(11):4855–64. 10.1364/BOE.8.004855 29188086 PMC5695936

[17] Wu X, Pine D, Chaikin P, Huang J, Weitz D. Diffusing-wave spectroscopy in a shear flow. Journal of the Optical Society of America B. 1990;7(1):15–20.

[18] Hovav T, Yedgar S, Manny N, Barshtein G. Alteration of red cell aggregability and shape during blood storage. Transfusion. 1999;39(3):277–81. 10.1046/j.1537-2995.1999.39399219284.x 10204590

[19] Risbano MG, Kanias T, Triulzi D, Donadee C, Barge S, Badlam J, et al Effects of aged stored autologous red blood cells on human endothelial function. American journal of respiratory and critical care medicine. 2015;192(10):1223–33. 10.1164/rccm.201501-0145OC 26222884 PMC4731619

[20] Kumar M, Dandapat S, Sinha MP, Kumar A, Raipat BS. Different blood collection methods from rats: A review. Balneo Research Journal. 2017;8(2):46–50.

[21] Ren K, Zhou J, Wu H. Materials for microfluidic chip fabrication. Accounts of chemical research. 2013;46(11):2396–406. 10.1021/ar300314s 24245999

[22] Brake J, Jang M, Yang C. Analyzing the relationship between decorrelation time and tissue thickness in acute rat brain slices using multispeckle diffusing wave spectroscopy. Journal of the Optical Society of America A. 2016;33(2):270–5. 10.1364/JOSAA.33.000270 PMC4783160 26831778

[23] Song S-H, Lim C-S, Shin S. Migration distance-based platelet function analysis in a microfluidic system. Biomicrofluidics. 2013;7(6):064101. 10.1063/1.4829095 PMC3838424 24396535

[24] Song S-H, Lim C-S, Shin S. Scalable evaluation of platelet aggregation by the degree of blood migration. Applied Physics Letters. 2013;103(24):243702.

[25] Baskurt OK, Meiselman HJ. Erythrocyte aggregation: basic aspects and clinical importance. Clinical hemorheology and microcirculation. 2013;53(1–2):23–37. 10.3233/CH-2012-1573 22975932

[26] Ljung GM, Box GE. On a measure of lack of fit in time series models. Biometrika. 1978;65(2):297–303.

[27] Baskurt OK, Uyuklu M, Ulker P, Cengiz M, Nemeth N, Alexy T, et al Comparison of three instruments for measuring red blood cell aggregation. Clinical hemorheology and microcirculation. 2009;43(4):283–98. 10.3233/CH-2009-1240 19996518

[28] Bauersachs R, Wenby R, Meiselman H. Determination of specific red blood cell aggregation indices via an automated system. Clinical hemorheology and microcirculation. 1989;9(1):1–25.

[29] Klose H, Volger E, Brechtelsbauer H, Heinich L, Schmid-Schönbein H. Microrheology and light transmission of blood. Pfluegers Archiv. 1972;333(2):126–39. 10.1007/bf00586912 4538028

[30] D’Alessandro A, Liumbruno G, Grazzini G, Zolla L. Red blood cell storage: the story so far. Blood Transfusion. 2010;8(2):82 10.2450/2009.0122-09 20383300 PMC2851210

[31] Lim H-J, Nam J-H, Lee B-K, Suh J-S, Shin S. Alteration of red blood cell aggregation during blood storage. Korea-Australia Rheology Journal. 2011;23(2):67–70.

[32] Almac E, Ince C. The impact of storage on red cell function in blood transfusion. Best practice & research Clinical anaesthesiology. 2007;21(2):195–208. 17650772 10.1016/j.bpa.2007.01.004

[33] Puniyani RR. Clinical hemorheology: new horizons: New Age International; 1996.

[34] Boas DA. Diffuse photon probes of structural and dynamical properties of turbid media: theory and biomedical applications: Ph.D. dissertation, University of Pennsylvania, 1996.

[35] Xu Z, Zheng Y, Wang X, Shehata N, Wang C, Sun Y. Stiffness increase of red blood cells during storage Microsystems & Nanoengineering. 2018;4:17103.

[36] Huang S, Hou HW, Kanias T, Sertorio JT, Chen H, Sinchar D, et al Towards microfluidic-based depletion of stiff and fragile human red cells that accumulate during blood storage. Lab on a Chip. 2015;15(2):448–58. 10.1039/c4lc00768a 25406942 PMC4268274

[37] Shin S, Yang Y, Suh J-S. Measurement of erythrocyte aggregation in a microchip stirring system by light transmission. Clinical hemorheology and microcirculation. 2009;41(3):197–207. 10.3233/CH-2009-1172 19276517

[38] Fine I, Fikhte B, Shvartsman LD, editors. RBC-aggregation-assisted light transmission through blood and occlusion oximetry. Controlling Tissue Optical Properties: Applications in Clinical Study; 2000;4162:130–139.

[39] Shin S, Jang J, Park M, Ku Y, Suh J. Light-transmission aggregometer using a vibration-induced disaggregation mechanism. Review of scientific instruments. 2005;76(1):016107.

[40] Bauersachs R, Wenby R, Pfafferott C, Whittingstall P, Meiselman H. Determination of red cell deformation via measurement of light transmission through RBC suspensions under shear. Clinical Hemorheology and Microcirculation. 1992;12(6):841–56.

[41] Chien S. Determinants of blood viscosity and red cell deformability. Scandinavian Journal of Clinical and Laboratory Investigation. 1981;41(sup156):7–12. 10.3109/00365518109097424 6948403

[42] Hardeman M, Dobbe J, Ince C. The Laser‐assisted Optical Rotational Cell Analyzer (LORCA) as red blood cell aggregometer. Clinical hemorheology and microcirculation. 2001;25(1):1–11. 11790865

[43] Armstrong JK, Wenby RB, Meiselman HJ, Fisher TC. The hydrodynamic radii of macromolecules and their effect on red blood cell aggregation. Biophysical journal. 2004;87(6):4259–70. 10.1529/biophysj.104.047746 15361408 PMC1304934

[44] Gautam R, Oh J-Y, Marques MB, Dluhy RA, Patel RP. Characterization of Storage-Induced Red Blood Cell Hemolysis Using Raman Spectroscopy. Laboratory medicine. 2018. 10.1093/labmed/lmy018 PMC6180846 29893945

[45] Chien S, Jan KM. Red cell aggregation by macromolecules: roles of surface adsorption and electrostatic repulsion. Journal of supramolecular structure. 1973;1(4‐5):385–409. 4358445 10.1002/jss.400010418

[46] Karon BS, Van Buskirk CM, Jaben EA, Hoyer JD, Thomas DD. Temporal sequence of major biochemical events during blood bank storage of packed red blood cells. Blood Transfusion. 2012;10(4):453 10.2450/2012.0099-11 22507860 PMC3496226

