# Supplementary figures and images for: Optofluidic laser speckle image decorrelation analysis for the assessment of red blood cell storage
Source: PLoS One. 2019 Oct 22;14(10):e0224036. doi: 10.1371/journal.pone.0224036 (PMC6805004; doi:10.1371/journal.pone.0224036)

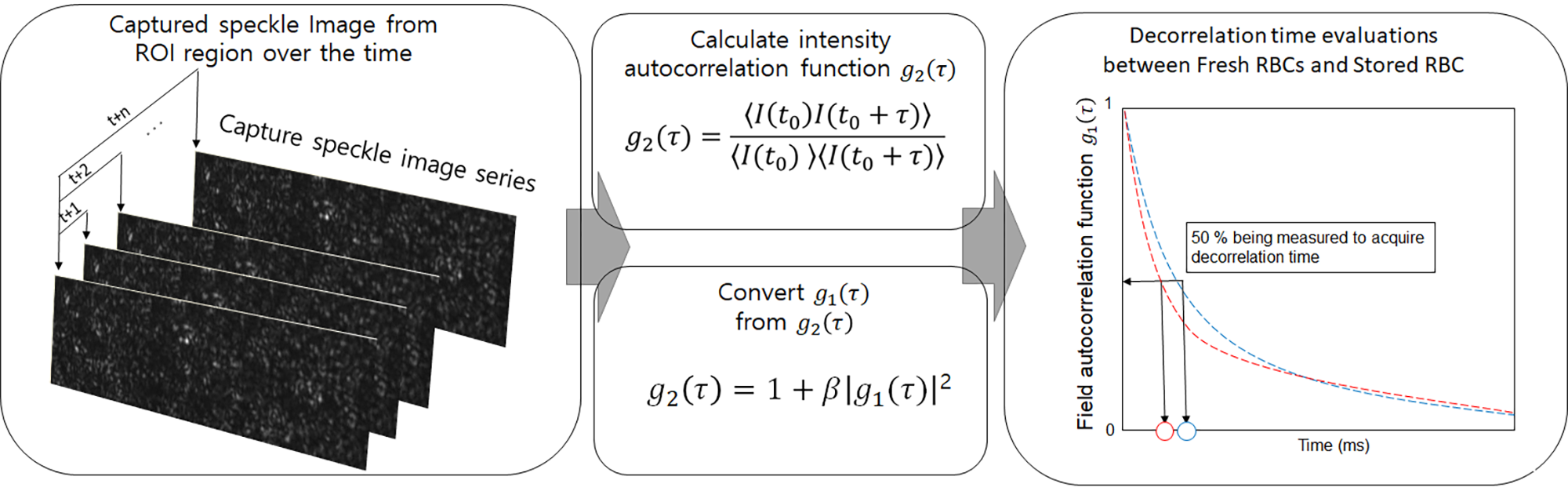

Supplement: S1 Fig — The sequence of speckle images over time was used for calculating the field autocorrelation function (g1(τ)). Decorrelation time is defined as the time for a correlation between the initial image and the subsequently captured image to drop by 50%. (TIF) [file pone.0224036.s003.tif]

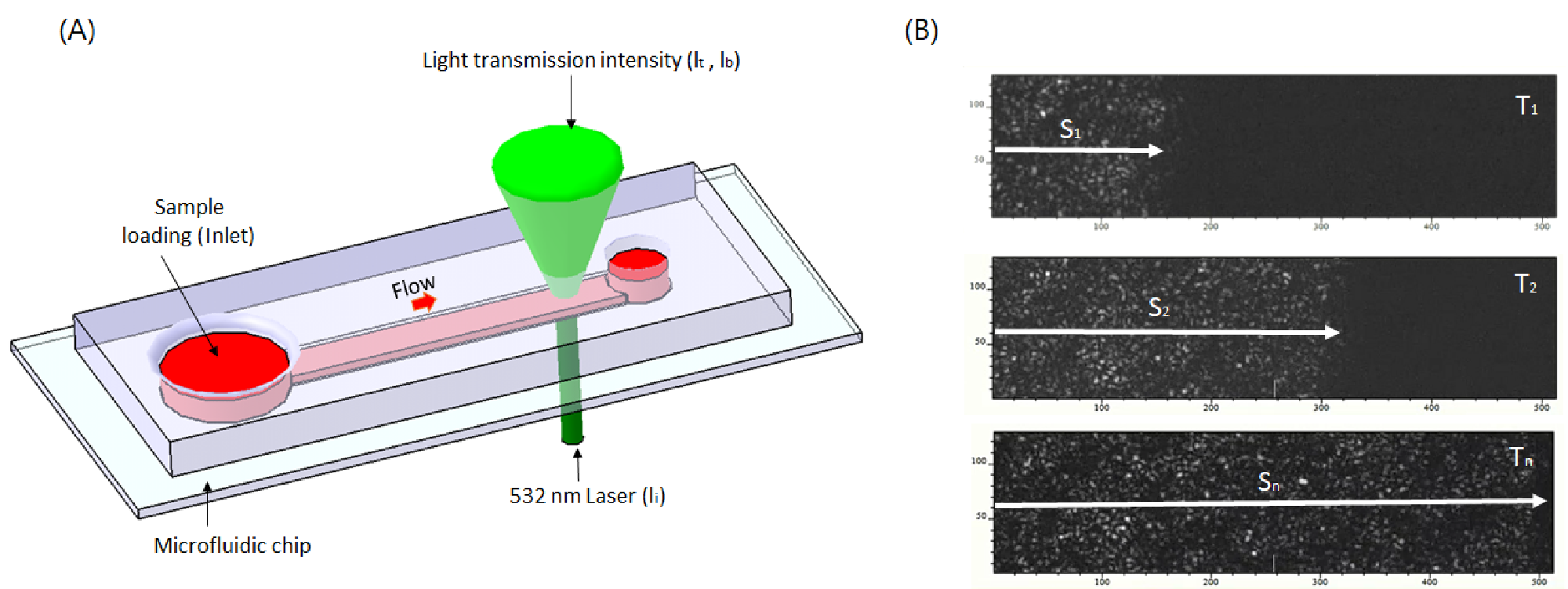

Supplement: S2 Fig — (A) shows the schematic for measuring relative light transmission intensity (li is initial light intensity, lt shows the transmitted light intensity passing through the channel filed with blood sample, and lb is the background light intensity passing through the channel unfilled with samples. (B) RBC flow speed was calculated using average speed v = Sn/Tn. (TIF) [file pone.0224036.s004.tif]

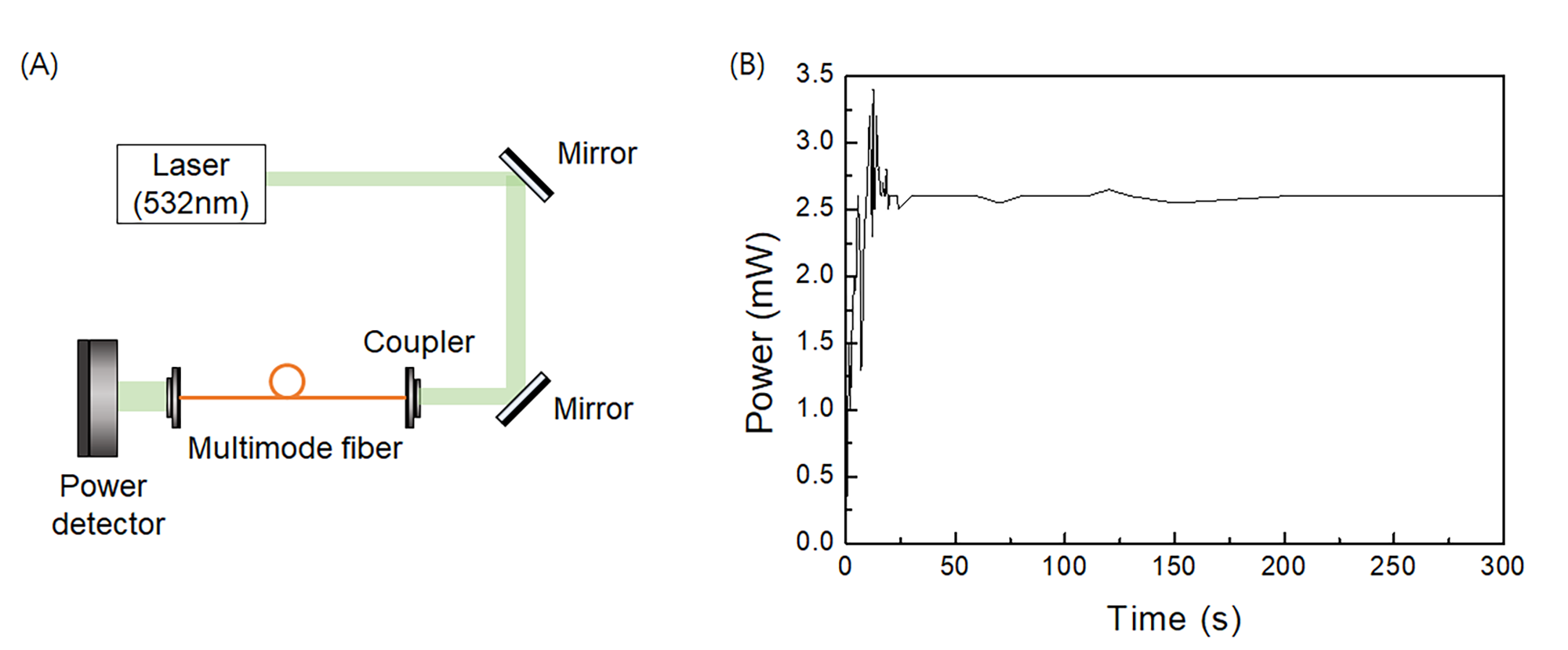

Supplement: S3 Fig — The schematic of setup to measure laser power fluctuations over time (A) and the acquired data (B). (TIF) [file pone.0224036.s005.tif]

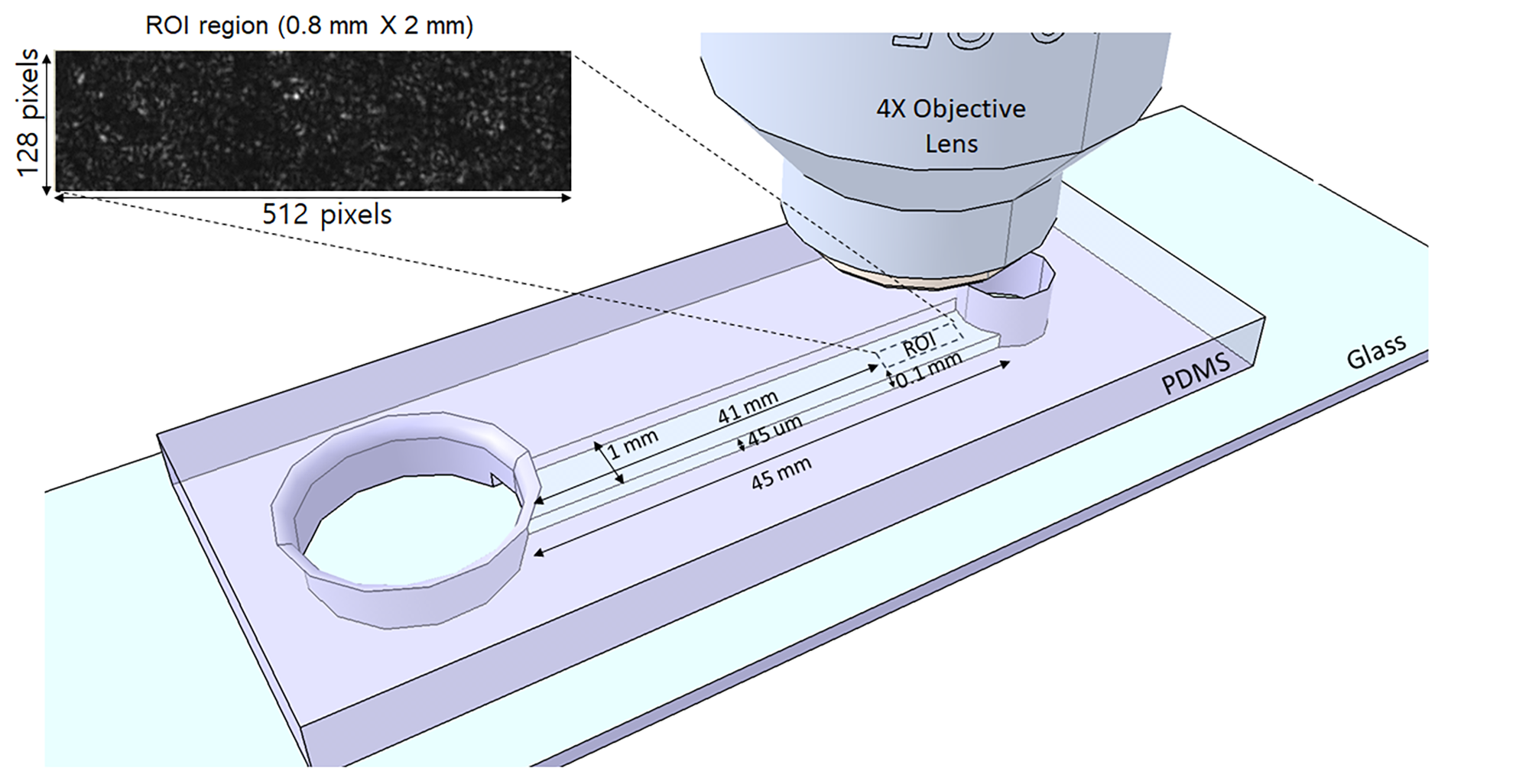

Supplement: S4 Fig — (TIF) [file pone.0224036.s006.tif]

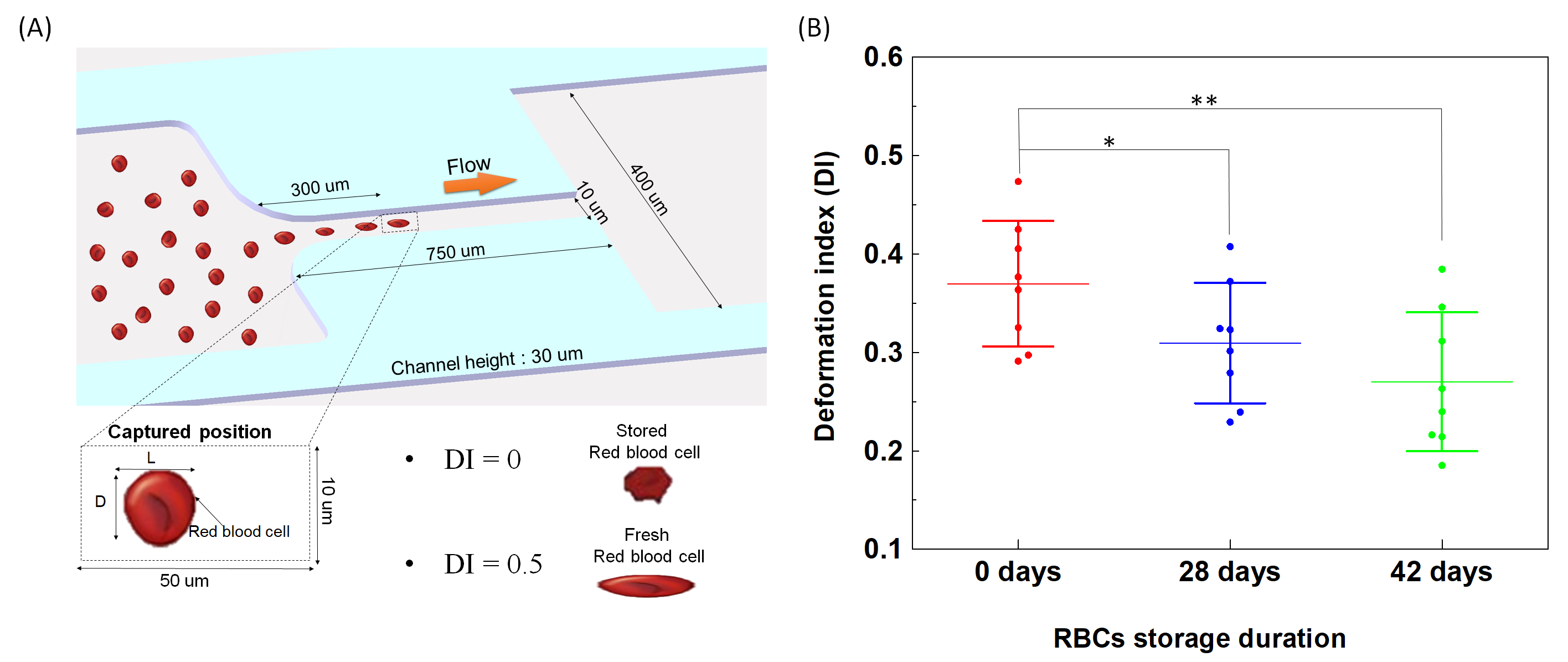

Supplement: S5 Fig — (A) Schematic diagram of the microfluidic device to measure RBC deformation index (DI). Individual RBCs are flowing through the channel undergoing shape changes. (B) RBC deformation indices over storage duration. For measurement of the stored RBC deformability, microfluidic devices was prepared through a typical photolithographic process. Treated blood sample of RBC suspension with 1% hematocrit, in PBS-albumin buffer, was inserted into the flow chamber. Image sequences of RBCs in the channel were obtained by a high-speed camera (Neo 5.5 sCMOS, Andor Technology Ltd. Belfast, UK). The deformation index (DI = L−DL+D) was calculated from the image sequences (L = axial RBC length, D = lateral RBC lengh). DI distribution decreased over the duration of RBC cold storage. High DI shows the highly deformable cell as close to 1, and low DI shows the low deformability. *: p < 0.05 and ** p < 0.01, ***: p < 0.001. (TIF) [file pone.0224036.s007.tif]

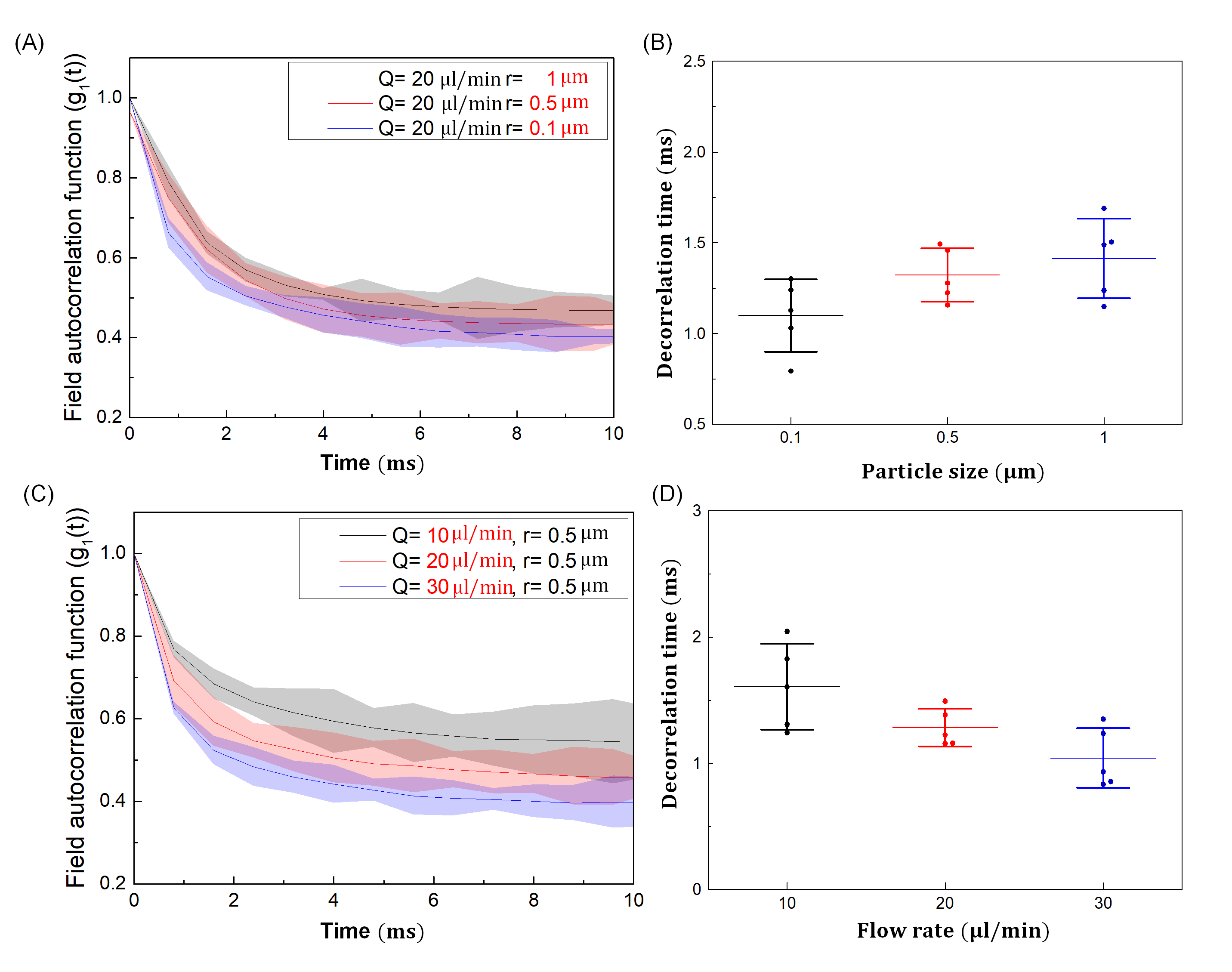

Supplement: S6 Fig — In Figure (A) and (C,) field autocorrelation function curves were calculated by capturing each speckle sequence by comparing the original reference frame. (B) shows decorrelation time was changing from different particle size. (C) show decorrelation time changing from the different flow rate. Particles concentration was prepared from diluted deionized water 3:10 (30% of particle with 1 μm = 12.1 × 109 particles/mL. Each groups decorrelation time were 1.10 ± 0.18 ms, 1.32 ± 0.14 ms and 1.41 ± 0.19 ms respectively in figure(B) and 1.61 ± 0.30 ms, 1.28 ± 0.13 ms, and 1.04 ± 0.21 ms in figure (D). (TIF) [file pone.0224036.s008.tif]
